# Opportunistic salpingectomy for prevention of ovarian cancer among the general population

**DOI:** 10.1097/GCO.0000000000001051

**Published:** 2025-07-22

**Authors:** Kirstie McLoughlin, Melissa A. Merritt

**Affiliations:** 1 The Daffodil Centre, The University of Sydney, A Joint Venture with Cancer Council NSW, Sydney, New South Wales, Australia

**Keywords:** guidelines, ovarian cancer, prevention, risk-reducing surgery, salpingectomy

## Abstract

**Purpose of review:**

Ovarian cancer remains the most lethal gynaecological cancer. Early detection and treatment options are limited, so prevention is key. This article reviews the current opinion on opportunistic salpingectomy for ovarian cancer prevention within the general population.

**Recent findings:**

Salpingectomy (the removal of the fimbriated ends of the fallopian tubes) reduces ovarian cancer risk without inducing early menopause or depleting ovarian reserves. The International Federation of Gynecology and Obstetrics firmly supports the use of salpingectomy opportunistically (in addition to planned abdominal surgery) for ovarian cancer risk reduction. When salpingectomy should be provided as an elective surgery, and what nongynecological surgeries can be used as opportunities for salpingectomy, is an ongoing discussion.

**Summary:**

As understanding of the safety and efficacy of opportunistic salpingectomy has solidified into guidelines, its use for ovarian cancer risk reduction has increased. However, the uptake of opportunistic salpingectomy has varied between geographic regions and across institutions. In the US, roughly a third of women undergo a hysterectomy during their lifetime, each of these women should have a chance to consider opportunistic salpingectomy for ovarian cancer prevention. Education about the benefits and harms of opportunistic salpingectomy is both necessary and effective in reducing inequities in access to this risk-reducing surgery.

**KEY POINTS**

- There is wide interest in diagnosing ovarian cancer at an early stage, but based on current evidence, screening for ovarian cancer in the general population is not recommended.
- Salpingo-oophorectomy has been used in women with high hereditary risk of ovarian cancer, but is not recommended in women at average risk because of the increased mortality risks from early menopause. In contrast, salpingectomy alone reduces ovarian cancer risk and does not appear to induce early menopause.
- After childbearing is completed, women at average risk of ovarian cancer should be supported to undertake salpingectomy when undergoing abdominal surgery (‘opportunistic salpingectomy’) to reduce their ovarian cancer risk.
- Thirteen gynaecological societies currently recommend opportunistic salpingectomy for the prevention of high-grade serous tubo-ovarian cancer in the general population. The International Federation of Gynecology and Obstetrics has released a position statement firmly supporting opportunistic salpingectomy.
- Opportunistic salpingectomy is cost-effective. Further research into what surgeries are appropriate for the addition of salpingectomy and when salpingectomy should be offered as an elective surgery is necessary.

## INTRODUCTION

Globally, ovarian cancer is the sixth most common cancer in women and the fifth most common cause of cancer death [1,2]. Despite advances in treatment, the 5-year relative survival for invasive epithelial ovarian cancer between 2012 and 2018 was 50% [3]. The large-scale UK Collaborative Trial of Ovarian Cancer Screening (UKCTOCS) clinical trial assessed screening strategies (transvaginal ultrasound and measurements of CA-125 levels with screening every 3–12 months) over a period of 5–10 years and followed up for ovarian cancer mortality for 15–20 years [4]. Although they observed shifts to a lower stage at ovarian cancer diagnosis in the trial, there was no improvement in overall survival. As a response to this important study, community and clinical interests have moved toward methods of prevention [5]. As most (c.80–90%) ovarian cancer cases appear to occur sporadically [6–8], preventive options in women at average risk of developing ovarian cancer are particularly important.

‘Salpingo-oophorectomy’, or the removal of the ovaries and the fallopian tubes, has been the standard of care for reducing ovarian cancer risk in women at high hereditary risk of developing ovarian cancer. These high-risk women are predominantly those with *BRCA1/BRCA2* pathogenic variants, though women with *RAD51* alterations or Lynch syndrome are also at increased lifetime risk [9]. Women at high risk of ovarian cancer are often identified through familial diagnoses or through genetic testing for pathogenic variants. Risk-reducing salpingo-oophorectomy has, historically, been restricted to women at high-risk of developing ovarian cancer as the removal of the ovaries results in early menopause, leading to increased risk of cardiovascular disease, and reducing all-cause survival in most women [10]; however, in women with known pathogenic *BRCA* variants (or other risk-increasing alterations) their lifetime ovarian cancer risk can be as high as 59% [9], which is considered to outweigh the risks associated with premature menopause.

In contrast, in the general population, the lifetime risk of developing ovarian cancer is less than 2% [11], and thus the health risks of early menopause are considered to outweigh the benefits of risk-reducing salpingo-oophorectomy for ovarian cancer prevention [10]. Instead, there has been long standing interest in screening for early detection of ovarian cancer within the general population; however, as noted above, large scale screening trials [4,12,13] have been unable to demonstrate improved survival resulting from currently available ovarian cancer screening strategies and therefore the US Preventative Screening Task Force does not recommend screening for ovarian cancer in the general population [14]. These results have led to a community-driven shift in interest towards methods for the prevention of ovarian cancer [5].

### Salpingectomy prevents ovarian cancer

Ovarian cancer is a diverse disease; mostly of epithelial origin (c. 90%), though some develop from germ-cell or stromal tumors [15]. Epithelial ovarian cancer is itself heterogeneous with five major histological subtypes: high-grade serous, low-grade serous, clear cell, mucinous, and endometrioid [16]. The majority of epithelial ovarian cancer cases are high-grade serous tubo-ovarian cancers (c. 75%) [16], which largely appear to originate in the fallopian tubes [17].

Salpingectomy is the removal of the fimbriated ends of the fallopian tubes. In 2015, Bradley and Visco [18] summarized the evidence for prophylactic bilateral salpingectomy at the time of urogynecological surgery as a method of ovarian cancer risk reduction, what is now referred to as ‘opportunistic salpingectomy’, noting that ‘the overall impact that universal removal of the fallopian tubes would have on ovarian cancer rates remains speculative’. Since their review, several cohort studies [19–24] have demonstrated the utility of salpingectomy in preventing high-grade serous tubo-ovarian cancer and potentially some other ovarian cancer histopathological subtypes.

These cohort studies have been systematically reviewed by Kahn *et al*. [25], Masouleh *et al*. [26], and Tang *et al*. [27^■^]. The key findings from these systematic reviews were that there was no significant impact of salpingectomy on ovarian function or quality-of-life when performed in addition to hysterectomy or cesarean delivery or in replacement of tubal ligation, and that salpingectomy can significantly lower the risk of high-grade serous tubo-ovarian cancer (though likely not to the extent reported in Kahn *et al*. [28]). The meta-analysis by Tang *et al*. [27^■^], based on one cohort study and five case-control studies, indicated that women who had a salpingectomy procedure had a significantly reduced risk of ovarian cancer (odds ratio: 0.63, 95% confidence intervals: 0.45–0.89). Though it should be noted that the seminal paper by Hanley *et al*. [24], described in Masouleh *et al*. [26], was not included in Tang *et al.*’s [27^■^] meta-analysis. Hanley *et al*. [24] published a retrospective cohort analysis set in Canada (British Columbia) investigating ovarian cancer incidence in women who undertook salpingectomy in addition to hysterectomy (in comparison to hysterectomy alone) or as an alternative to tubal ligation and observed that women who had salpingectomy did not develop serous ovarian cancer and had fewer epithelial ovarian cancer cases overall.

Unlike salpingo-oophorectomy, salpingectomy does not appear to induce premature menopause [29]. Multiple studies have now demonstrated that salpingectomy does not increase the risk of perioperative adverse outcomes or minor complications when added to a surgical intervention [30–32]. Further studies have found no impact of salpingectomy on ovarian reserves [33,34]. Salpingectomy, particularly when performed opportunistically, changes the benefit/risk trade-off of risk-reducing surgery for the prevention of ovarian cancer in the general population.

### Guidelines for opportunistic salpingectomy

Since 2018, 13 International Federation of Gynecology and Obstetrics (FIGO)-affiliated national societies have published guidelines or position statements on opportunistic salpingectomy for ovarian cancer prevention [35]. In 2018, nine of the 13 national societies with statements recommended opportunistic salpingectomy, and four remained ambivalent (Germany, France, Norway, and Sweden). Currently, 11 of 13 recommend that salpingectomy should be performed as a method of preventing ovarian cancer when women are already undertaking urogynaecological surgery (updated guidelines for Germany [36] and France [37]), and FIGO has released a position statement that they ‘firmly support opportunistic salpingectomy’ [38^■■^]. Opportunistic salpingectomy is becoming standard practice even in countries that were historically ambivalent [39].

In brief, FIGO guidelines suggest clinicians provide comprehensive counseling on opportunistic salpingectomy. Salpingectomy at the time of gynecologic surgery is low risk, but the risk may be greater if salpingectomy is performed in addition to upper abdomen laparoscopies. Informed consent for opportunistic salpingectomy should include consulting women on the rationale for salpingectomy, the difference in function of the fallopian tubes and ovaries, and that salpingectomy is a method of surgical sterilization without menopausal onset. Women should also be informed of the elective nature of the procedure and any additional costs.

The uptake of opportunistic salpingectomy has changed over time. Even before the introduction of relevant guidelines, in 2011 in Canada [40] and in 2012/2015 in the US [35], the rates of opportunistic salpingectomy were observed to be generally increasing [31,41]. In the US, opportunistic salpingectomy rates rose from 2.4% in 2001 to 5.7% in 2010 and then leapt to 58.4% after the introduction of guidelines [41]. Since the introduction of guidelines, the rates of opportunistic salpingectomy have continued to increase; however, uptake of opportunistic salpingectomy is variable across regions and institutions [40,42,43]. Education about the benefits and harms of opportunistic salpingectomy is both necessary and effective; for example, uptake of opportunistic salpingectomy remains significantly higher in British Columbia (where an educational campaign was run in 2010 [31]) than in any other Canadian jurisdiction [43].

### Prevention of ovarian cancer in the general population is cost-effective

The increasing interest in preventive interventions for ovarian cancer has motivated studies using mathematical modeling methods (simulations) assessing the relative costs and benefits of risk-reducing surgery, including salpingectomy, alongside generalized genetic screening programs. The cost-effectiveness of risk-reducing surgical strategies has been systematically reviewed by Sroczynski *et al*. [44] and Wei *et al*. [45]. Most of these simulation studies have focused on women with high hereditary risk of developing ovarian cancer or their families (21 of the 33 studies reviewed in Sroczynski *et al*. and 15 of the 22 studies in Wei *et al*.). Since 2022, there have been a further eight cost-effectiveness analyses investigating preventive interventions for ovarian cancer [46–53], with three set in the general population [46–48], and seven simulation studies assessing genetic screening in the general population aimed at identifying women at high risk of ovarian cancer [54–61] (most often *BRCA* carriers [54,56,58–60]).

Among the three simulation studies focusing on preventive interventions in the general population, Hughes *et al*. [46] used a Markov model to demonstrate that opportunistic salpingectomy in women over 40 and opportunistic salpingo-oophorectomy in women over 50 were cost-effective compared with no intervention in the general population. This result was supported by Matsuo *et al*. [47], who similarly demonstrated that salpingectomy was cost-effective compared with no intervention at the time of laparoscopic cholecystectomy. Wagar *et al*. [48] showed that salpingectomy compared well with tubal ligation, with salpingectomy being clinically more effective in preventing pregnancy and reducing ovarian cancer cases, and cost-effective as an alternative to tubal ligation. Specifically, Wagar *et al*. demonstrated that salpingectomy increased costs manageably (incremental cost-effectiveness ratio of $26 149 per quality-adjusted-life-year) and improved outcomes (reducing ovarian cancer cases, ovarian cancer deaths, and increasing quality-adjusted-life-years) while also resulting in fewer unintended pregnancies than tubal ligation.

### Considering salpingectomy as a routine surgery for ovarian cancer prevention

Opportunistic salpingectomy is a cost-effective intervention that lowers ovarian cancer risk; however, the assessment of the relative benefits and harms of salpingectomy as an elective surgery to reduce ovarian cancer risk is just starting [62,63^■^]. Currently, approximately a third of women in the US undergo hysterectomy over the course of their lives [64], but recent data shows a decline in hysterectomy rates over time [65,66], which suggests that there may be fewer opportunities for women to receive salpingectomy opportunistically in the future. Moreover, hysterectomy rates tend to vary by access to healthcare, geographical region, and between racial and ethnic groups [65,67,68]; thus there is potential for inequities in access to salpingectomy as an opportunistic risk-reducing surgery [69].

The discussion around opportunistic salpingectomy has moved from gynaecological surgeries to assessments of other abdominal surgeries (e.g. cholecystectomy) and the training requirements for nongynaecological surgeons to incorporate salpingectomy into their practice [62,70^■^]. The cost-effectiveness analyses from Hughes *et al*. [46] and Matsuo *et al*. [47] have assessed the addition of salpingectomy to cholecystectomy and nongynecological elective procedures positively, and this discussion is further summarized in Verhoeff *et al*. [70^■^]. Studies of risk thresholds for interventions in premenopausal and postmenopausal women have, to date, only assessed salpingo-oophorectomy [71,72]. In these hypothetical cohorts, salpingo-oophorectomy was cost-effective for ovarian cancer risk reduction in postmenopausal women at 5% lifetime risk of ovarian cancer and in premenopausal women at 6% lifetime risk. Future studies are needed to extend these assessments to evaluate the risk thresholds for salpingectomy as an elective surgery.

## CONCLUSION

In the US, roughly a third of women undergo a hysterectomy over their lifetime [64], a fifth have pelvic floor surgery [73], and nearly 40% of women over the age of 40 years use tubal ligation as a form of contraception [74]. In line with current guidelines, each of these interventions provides a chance to consider opportunistic salpingectomy for epithelial ovarian cancer prevention. Opportunistic salpingectomy does not increase the risk of surgical complications, and it also does not appear to induce early menopause. Uptake of opportunistic salpingectomy has not been uniform, and educational campaigns targeting clinicians can help to provide patients with information to make informed decisions about adding salpingectomy to their surgery. As the rates of tubal ligation in the US are increasing [75] (by 3% each month from July to December 2022, following the Dobbs decision in banned states), it is important to educate clinicians and patients about the relative benefits of salpingectomy as an alternative to tubal ligation [76,77] or as an addition to abdominal surgery [78], to reduce their risk of ovarian cancer.

## Acknowledgements

K.M. and M.A.M. conceptualized this review, reviewed, edited, and approved the final version of the manuscript. K.M. reviewed the literature and wrote the original draft manuscript.

All data included within this review can be found in the manuscript or the original publications referenced.

## Financial support and sponsorship

This work was funded by the Fussell Family Foundation. M.A.M. is supported by a Department of Defense Ovarian Cancer Research Program, Ovarian Cancer Academy Early Career Investigator Award (OC200236, W81XWH-21-1-0914). Opinions, interpretations, conclusions, and recommendations are those of the authors and are not necessarily endorsed by the Department of Defense or other funding bodies. The funders had no role in the study design, analysis, interpretation, or manuscript preparation.

### Conflicts of interestThere are no conflicts of interest.
